# Vaccination and Small-Pox in the United States Army

**Published:** 1899-08-12

**Authors:** 


### VACCINATION AND SMALL-POX IN THE UNITED STATES ARMY.

A report has recently been made to the United States Secretary for War in reference to the prevalence of small-pox in the United States armies in Cuba and Manila. It appears that the intention of the regulations in force is to afford protection against small-pox to every man who is enlisted by a careful vaccination at the time of his entry into the service. Under these regulations, which have been faithfully carried out, the soldiers of the United States regular army have received efficient protection from the disease, for although it has prevailed in many parts of the country during the past fifteen years—1883-1897—and frequently with epidemic violence, among the civil population in the immediate vicinity of military posts, there have only occurred twenty cases, of which four were fatal, in a mean strength of 25,000 men. At the outbreak of the Spanish-American war a large volunteer force was hastily organised, and the regiments of the regular army were as hastily recruited to their full strength on a war footing, the army being thus in a few weeks increased from 25,000 to 280,000 men. Under these circumstances it is possible that the regulations as to vaccination were not as carefully fulfilled as in time of peace, for it is to be observed that according to these regulations the necessity for revaccination is left to the decision of the medical officer. Now it was known that small-pox had been more or less prevalent among the coloured population of the Southern States, and also that it was common in Cuba, and it seems probable that this knowledge acted as a stimulus to careful carrying out of the regulations among the troops recruited in the Eastern States, whereas those recruited in the west did not anticipate immediate exposure to infection. At any rate what happened was that scarcely any cases occurred among the troops employed in Cuba and Porto Rico, although many of them were actively engaged in stamping out the disease, which at the time was decimating the native population. On the other hand a number of cases appeared among certain regiments soon after their arrival at Manila, these cases being mostly among the men who had been visiting the huts of the natives, in many of which small-pox of a very malignant character was then prevailing. However, immediate steps were taken to prevent the spread of the disease. Every enlisted man who had not been successfully vaccinated within six months was required to be vaccinated at once, and it was ordered that the operation should be repeated if unsuccessful; more care also was exercised in regard to the vaccination of all men sent out, and the result has been that the outbreak among the troops is at an end, and that the army in Manila may now be considered to be protected. From September, 1898, to March, 1899, however, there were 151 cases of variola, with 77 deaths, and 85 cases of varioloid with no death, among the 30,000 men in the command. We have in this only another example of the efficacy of vaccination in controlling epidemic small-pox, and also of the dangers of putting faith in vaccination which may have been imperfectly performed.

